# The Salt of Their Profession

**Published:** 1913-01-11

**Authors:** 


### THE EDITOR'S LETTER-BOX.

### The Salt of their Profession.

Let honour be to us as strong an obligation as necessity is to others.—*Pliny.*

*To the Editor of* The Hospital.

Sir,—I am a registered member of the medical profession, and as such concerned for the honour of the profession. I am of a family many men of which for many generations have practised as general practitioners. I am proud to say, without exception, all have been honoured and respected in and beyond the spheres of their work. Circumstances place me outside the need to consider the advisability of serving or not serving under the Insurance Act. I have made no agreement; I have taken no oath. I am an impartial observer of the difference between Mr. Lloyd George and the medical practitioners as far as it is possible for a medical man to be impartial. Economically I am not concerned; but the morality of a body of men is the concern of every member of that body. What is the morality of the medical profession now? How does the conduct of a majority of the medical profession look to an impartial observer? To be brutally frank, the position looks to be this: Thousands of medical men made a solemn promise each one to each other that he would not serve under the Insurance Act. This promise appears to have been made by two sorts of men: men who having given their word stand by it; and men who having given their word stick to it only if it pays to stick to it. Unfortunately, to the general public it must appear that the most of the oath-swearers of the medical profession are of the latter class. Is it good for the medical profession that the public should infer, as the public must, that the most of medical men do not feel bound to keep plighted troth if keeping plighted troth touches the pocket? For that is seemingly the position. The men swore solemnly and with thought, when they thought it would advantage them, that they would not serve under the Act. Readily they broke their oaths so soon as they thought they would lose money by keeping their word, or when they thought they could increase their incomes by breaking it. "Under economic pressure," I believe, is the formula of excuse.

There are still qualified medical men who, having pledged their word, are keeping their word. On these men pressure is being brought by the Government of Great Britain to force them to break their word. May the attempt fail! The English people by their representatives in Parliament are penalising the only honourable men of the medical profession for honourably abiding by their pledge. The people of England are taking from these men, whose only fault is that they abide by their word, appointments such as the medical care of post-office employés for no other reason than that these men have not broken their oaths, and will keep their oaths until formally absolved, and brother-members of an "honourable" profession are scrambling for the money of which these men are deprived.

Can it be well with Britain when her Government persecutes the honourable and rewards the unworthy? Can it be well with the medical profession when the most of its members do not keep troth? Will not insured persons recognise that most of the men who will attend them, so far from being honourable men, could not be on the panel had they not sold honour for gold?—I am, Sir, etc.,

January 8, 1913. L.R.C.P., M.R.C.S.

*Honour before all.*

"Be and continue poor, young man, while others around you grow rich by fraud and disloyalty; be without place or power, while others beg their way upward; bear the pain of disappointed hopes, while others gain the accomplishment of theirs by flattery; forgo the gracious pressure of the hand, for which others cringe and crawl. Wrap yourself up in your own virtue, and seek a friend and your daily bread. If you have, in such a course, grown grey with unblenched honour, bless God and die."—*Heinzelmann.*

